# A Robust Infrared Transducer of an Ultra-Large-Scale Array

**DOI:** 10.3390/s20236807

**Published:** 2020-11-28

**Authors:** Defang Li, Jinying Zhang, Qingfeng Shi, Xichen Yuan, Zhuo Li, Xin Wang, Suhui Yang, Yan Hao

**Affiliations:** 1 School of Optics and Photonics, Beijing Institute of Technology, Beijing 100081, China; 3120195347@bit.edu.cn (D.L.); 81908027@bit.edu.cn (Q.S.); 3220180387@bit.edu.cn (X.Y.); lizhuo@bit.edu.cn (Z.L.); wangxin@bit.edu.cn (X.W.); suhuiyang@bit.edu.cn (S.Y.); 3120195302@bit.edu.cn (Y.H.); 2 Beijing Key Laboratory for Precision Optoelectronic Measurement Instrument and Technology, Beijing 100081, China

**Keywords:** infrared transducer, infrared image generation, ultra-large-scale array, silicon micro cavities

## Abstract

A robust micro-electro-mechanical systems (MEMS) infrared thin film transducer of an ultra-large-scale array was proposed and fabricated on a 4-inch silicon wafer. The silicon substrate and micro cavities were introduced. This novel transducer had excellent mechanical stability, time response, and state-of-the-art pixel scale. It could bear a load of 1700 g and its load pressure was improved by more than 5.24 times and time constant decreased by 50.7% compared to the traditional soft infrared thin film transducer. The array scale of its pixels exceeded 2k × 2k. The simulation and measured results of the transient temperature and radiation intensity were well consistent. Illuminated by a 532 nm laser with a frequency of 50 Hz and 50% duty cycle, the thermal decay time of the proposed transducer was 6.0 ms. A knife-edge image was utilized for spatial resolution test and the full width at half maximum (FWHM) of the proposed transducer was 24% smaller than the traditional soft one. High-resolution infrared images were generated using the proposed robust transducer. These results proved that the robust transducer was promising in infrared image generation.

## 1. Introduction

The past decade has witnessed the rapid development of unmanned driving technology. The key to the breakthrough of unmanned driving technology lies in the safe driving under various uncertain conditions [1]. Besides driving safely in the daytime, driverless vehicles also need to drive safely in poor visibility, such as in dark night or bad weather. The recognition of targets (cars, people, and obstacles) and road background in non-visible light band needs the assistance of microwave radar and infrared thermal imaging [2]. Infrared imaging benefits higher detection resolution than microwave imaging, and various infrared sensors and detectors have achieved great progress in recent years [3,4,5,6,7]. Consequently, it gains considerable attention and has grown rapidly in unmanned driving technology [8]. To enhance the ability of target recognition, numerous infrared images and videos are required for target recognition training. A direct way to obtain numerous infrared images is to image the targets in the real world. However, it is quite difficult to acquire numerous infrared images from the real world under severe weather conditions, such as heavy fog, strong wind, blowing sand, heavy snow, freezing, hail, and heavy rainfall, etc. Target recognition in severe weather conditions is of vital importance for the safety of unmanned driving system. Thus, it is critical to generate the infrared images of targets in bad weather.

Infrared image generation technology provides a promising solution to this problem. Infrared image generation technology can simulate the infrared radiation characteristics of targets and scenes, and provide high-quality infrared radiation source for infrared detector [9]. It will dramatically reduce costs and time to acquire the target infrared images of severe weather conditions. In addition, infrared imaging can also simulate the infrared characteristics of moving targets and backgrounds [10], so it can also assist driving decision simulation test as well as infrared imaging guidance training [11].

According to the generating method, infrared image generation technology can be divided into two categories: direct radiation technology and modulated radiation technology. Direct radiation infrared image generation technology can directly produce infrared radiation through the electro-optical conversion (such as light emitting diode [12], laser diode array [13], etc.), electro-thermal-optical conversion (such as resistor array [14,15,16]), and optical-thermal-optical conversion (such as MEMS infrared thin film transducer [17,18,19]). Modulated radiation infrared image generation technology (such as Digital Micromirror Devices, DMD [20,21]; IR liquid crystal light valve [22], MEMS optical attenuator [23], etc.) can modulate the intensity distribution by changing the reflection or transmission of the infrared radiation. Among these technologies, light emitting diode needs ultra-low operating temperature (77 K) and laser diode array suffers from severe deposition heat and narrow radiation band [24,25]. IR liquid crystal light valve has limitations in frame rate, temperature range and dynamic range [26]. MEMS optical attenuator array has the limitations of small array size and low frame rate [27,28,29,30]. Presently, the widely used infrared image generation technologies include DMD, resistor array and MEMS infrared thin film transducer. Table 1 presents the comparison of widely used infrared image generation technologies.

DMD is restricted by diffraction in the long wave IR band although it can achieve desired frame rate [31,32]. Limited by the complicated read-in integrated circuits, the maximum pixels of single resistor array are 512 × 512. Large scale resistor array is often assembled by several single resistor arrays, which leads to more complicated TSV (through silicon vias) and QP (quilt packaging) processes [16]. By contrast, MEMS infrared thin film transducer makes full use of the mature visible light imaging technology. It absorbs the visible light, and its temperature rises to radiate the infrared ray. Therefore, it could transduce visible light energy to infrared ray energy. Without requirement of read-in integrated circuits, the MEMS infrared thin film transducer can achieve larger scale pixels. It has been fabricated into 1313 × 1313 pixels and covers the wavelength range of 3~12 μm [17,18,19]. However, because this transducer is a soft composite film suspended on a large solid ring and its thickness is only several micrometers, it suffers from poor mechanical stability. During installation and use, it tends to be damaged easily. This damage is prominent when the pixel increases.

In this paper, we proposed a robust MEMS infrared thin film transducer based on silicon micro cavity structure. Different from the traditional soft one, the proposed transducer consisted of composite film suspended on an array of silicon micro cavities instead of a large ring. Using MEMS processing technology, we achieved more than 2000 × 2000 pixels on the entire 4-inch silicon wafer, which provided a wafer level infrared transducer of the most advanced pixels. The introduction of the silicon substrate increased the overall mechanical stability of the transducer, making it more robust and not easy to be damaged in the process of installation and use. Compared to the traditional soft infrared thin film transducer, its load pressure was improved by more than 5.24 times. The micro cavity structure on silicon substrate guaranteed good temperature and radiation characteristics of the MEMS infrared thin film. Illuminated by a 532 nm laser with 5.4 W/cm^2^ power density, the maximum radiation intensity was 1600 W/m^2^ and the temperature difference reached 171 K. A knife-edge image was utilized for resolution test and the full width at half maximum (FWHM) of the robust MEMS infrared thin film transducer was 24% smaller than the traditional soft infrared thin film transducer.

In addition, because of the high thermal conductivity of the silicon substrate, the time constant of the proposed robust transducer decreased by 50.7% than that of the traditional soft one. The time response improved with the decrease of time constant, which was necessary for generating the dynamic simulation infrared images.

For the infrared transducer, the refresh rate becomes higher when the time response gets faster. Because of the effect of human visual persistence, the refresh rate of 24 frames is sufficient to produce continuous images in human eyes. Therefore, for the infrared simulation test of unmanned driving, it is acceptable that infrared image generation technology can produce more than 24 frames of infrared dynamic images or videos. In order to improve the accuracy and reliability of the system, it would be better if the picture is smoother without dragging. This means the refresh rate should be more than 50 Hz (similar to the frequency of the projector). This proposed robust transducer was designed in this work to satisfy the frame rate of 50 Hz. Moreover, for some military applications, the target (aircraft, missile, etc.) movement rate is quite fast, and the refresh rate should be higher (some have exceeded 200 Hz [21]) to meet the specific requirements.

## 2. Structure and Principle of MEMS Infrared Thin Film Transducer

### 2.1. Structure of MEMS Infrared Thin Film Transducers

To enhance the mechanical stability, we designed and added a robust substrate to the traditional soft MEMS infrared thin film transducer. Here we selected silicon as the substrate because it benefited mature mass production technology. However, the introduction of silicon made it necessary to deliberately deal with thermal isolation issue.

Thermal isolation is a key factor in MEMS infrared transducers and the typical method to solve this problem is to introduce air holes as the isolation structure. Consequently, we need to etch the silicon substrate beneath the pixels to form air holes [4]. The popular etching process of silicon substrate includes surface micromachining on the front side [33] and bulk micromachining on the back side [34]. Taking into account the manufacturing difficulty and the undesired mechanical stability of bulk micromachining, we chose surface micromachining approach—etching silicon on the front side to form silicon micro cavities.

**Figure 1:**
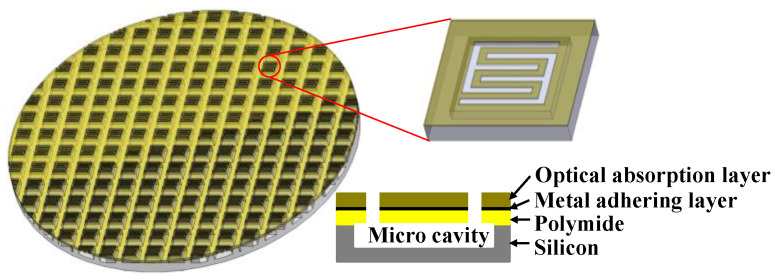
Schematic image of the proposed robust micro-electro-mechanical systems (MEMS) infrared thin film transducer with micro cavities.

**Figure 2:**
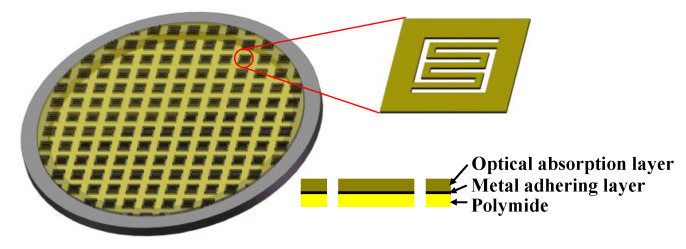
Schematic image of the traditional soft MEMS infrared thin film transducer.

**Figure 3:**
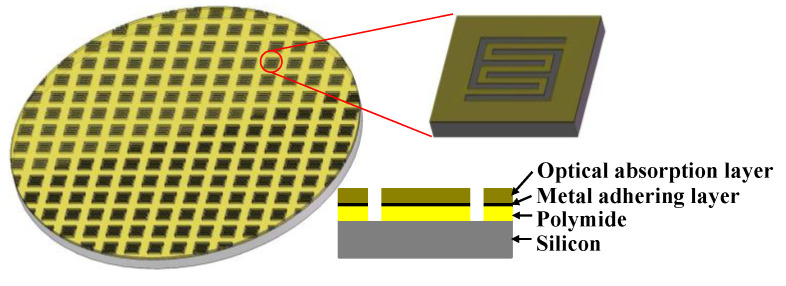
Schematic image of silicon-based MEMS infrared thin film transducer without micro cavities.

**Figure 4:**
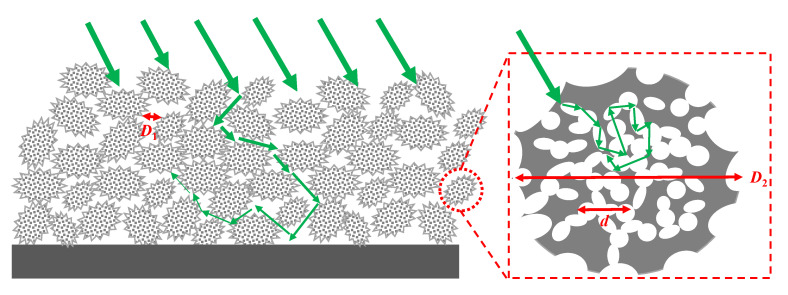
Absorption principle schematic of the metal black clusters.

Figure 1, Figure 2, Figure 3 present the MEMS infrared transducers. The robust MEMS infrared thin film transducer includes four parts, as shown in Figure 1. From top to bottom are optical absorption layer, metal adhering layer, polyimide (PI) layer, and silicon micro cavities. The optical absorption layer absorbs the incident visible light energy and the pixels are heated up to produce infrared radiation. The common absorption layer materials of MEMS infrared thin film transducer include metal black [18,35,36] and carbon nanotube composite materials [37]. The metal black was selected in this work because of much higher absorptivity (above 90% [18]) compared to that of carbon nanotube composite materials (about 60% [37]). The dimensions of the metal black clusters are critical to the absorption performance. As shown in Figure 4, the metal black cluster had a cotton-like structure. The gaps with critical dimension of *D*_1_, the clusters with critical diameter of *D*_2_, and the numerous tiny holes with critical dimension of *d* contributed together to multiple scattering of incident light whose wavelength was compatible to these critical dimensions. The atoms in the cluster absorbed the light energy, its kinetic energy increased, and the macro performance was that the temperature of the cluster rose. Consequently, the infrared ray was radiated. Researchers tried to build theoretical models to simulate the absorption properties of the metal black cluster [17]. We used a periodic nano-forest array to substitute the amorphous structure, and obtained simulation absorptivity well consistent with the measured results of the cotton-like clusters [17]. However, since the clusters varied in shapes and sizes, and they had numerous holes of varying shapes and sizes, the exact modelling of this complicated amorphous architecture was quite difficult. Researchers often chose to use the method of experiment and measurement to obtain the absorption properties of the cluster structure. In the experiments of our previous work [18], we found that as the diameter of evaporated metal black clusters increased from 50 nm to 300 nm, the absorption performance was improved by about 20% in the wavelength range of 400 nm to 800 nm. This was because as the diameters increased, the cluster performed a light-trapping effect in a broad visible light range. The diameter of the metal black cluster increased with the evaporation thickness. Thus, to ensure a relatively high absorptivity in the visible light range, the metal black layer should have a relatively large thickness. Because the optical absorption layer had poor adhesion, the metal adhering layer was adopted to combine the optical absorption layer and the PI layer. When the thickness of the metal adhering layer increased, the thermal mass and the time constant of the MEMS infrared thin film would also increase. This led to slower time response. Consequently, the thickness of the metal-adhering layer was controlled within 50 nm. PI layer was used as the supporting layer and it benefited much lower thermal conductivity than conventional supporting materials such as silicon dioxide and silicon nitride. Thus, it contributed to larger temperature difference which was necessary to obtain higher imaging contrast. Moreover, the in-plane thermal conductivity of the PI layer could be controlled by optimizing the PI thickness and patterns [38,39]. The single pixel could be designed into various patterns. To guarantee a high yield in wafer-level process in laboratory lithography, we set the minimum in-plane critical dimension as two micrometers.

For comparison, Figure 2 presents the structure of the traditional soft MEMS infrared thin film transducer. It includes the optical absorption layer, metal-adhering layer, and PI layer which are the same as the proposed robust one, while it does not have any silicon micro cavities nor silicon substrate beneath the pixels.

To investigate the effect of silicon micro cavities, we designed a contrast transducer using ordinary silicon wafer to replace silicon micro cavities wafer, as shown in Figure 3. It means the difference between the proposed robust transducer and the contrast one lies only in the silicon micro cavities.

Thus, three kinds of MEMS infrared thin film transducers were fabricated and compared. The first was the proposed robust one with silicon micro cavities structure (Figure 1), the second was the traditional soft one (Figure 2), and the third was the robust one without silicon micro cavity structure (Figure 3).

### 2.2. Principle of Robust MEMS Infrared Thin Film Transducer

**Figure 5:**
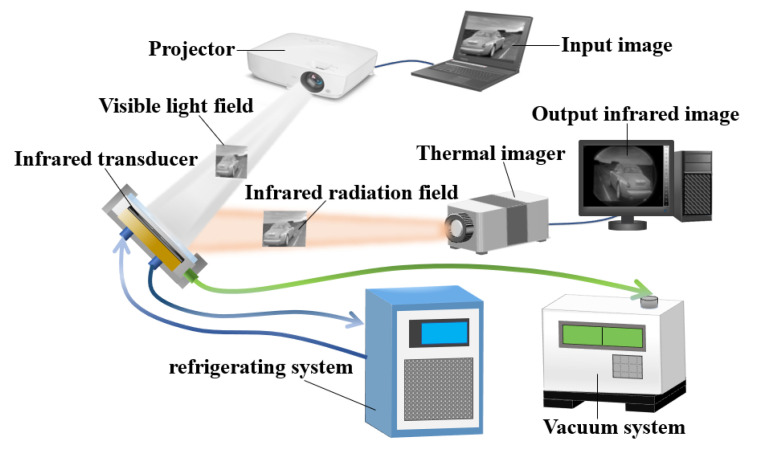
Working schematic of the proposed robust MEMS infrared thin film transducer.

According to the film heat conduction theory, when the visible light with a certain light energy distribution irradiates the surface of the robust MEMS infrared thin film transducer, different positions of the film surface will absorb different amounts of light energy and produce the corresponding temperature field distribution. As shown in Figure 5, the incident visible light is a gray image, different gray levels carry different visible light energy and the resulting infrared light field also has corresponding energy distribution. The heat conduction equation of thin films can be described as [40,41]:

(1) $$\rho dc_{p}\frac{\partial T}{\partial t}=\alpha q+kd\frac{\partial^{2}T}{\partial {\vec{r}}^{2}}-\sigma \varepsilon \left(T^{4}-T_{\mathrm{amb}}^{4}\right)-h\left(T-T_{\mathrm{amb}}\right)$$

where *ρ* is the density of the MEMS infrared thin film, *d* is the thickness of the MEMS infrared thin film, *c_p_* is the specific heat capacity of the MEMS infrared thin film, *T* is the actual temperature of the MEMS infrared thin film, *α* is the absorptivity of the MEMS infrared thin film, *q* is the power density of incident light, *k* is the thermal conductivity of the MEMS infrared thin film, *σ* is the Stefan-Boltzmann constant of 5.67 × 10^−8^ W/(m^2^∙K^4^), *ε* is the emissivity of the MEMS infrared thin film, *h* is the convective heat transfer coefficient, *T*_amb_ is the ambient temperature. Here the ambient temperature is usually taken as the air temperature in the vacuum chamber.

The left term of the equation indicates that the temperature of robust MEMS infrared thin film changes with time and is related to the density, specific heat capacity, and thickness of the MEMS infrared thin film. For the steady-state heat conduction equation, the left term is 0. The right term of the equation presents the absorption, conduction, convection, and radiation of the robust MEMS infrared thin film. The first item on the right side of Equation (1) is the visible light power density absorbed by the MEMS infrared thin film, which is related to the absorption rate of the film and incident light power density; the second item is the three-dimensional heat conduction term, which is related to the thermal conductivity and thickness of the MEMS infrared thin film in three dimensions; the third item is the radiation heat transfer power density between the surface of the MEMS infrared thin film and the environment, which is related to the emissivity of the film surface and the ambient temperature; the fourth item is the power density of convective heat transfer between film surface and air, which is related to convective heat transfer coefficient and air temperature. In order to reduce the convective heat transfer between the film surface and the air, the robust MEMS infrared thin film usually works in a high vacuum environment, so the fourth item can be neglected.

Figure 5 shows the working schematic of the proposed robust MEMS infrared thin film transducer. A gray input image was incident to the MEMS infrared thin film transducer by a visible light projector. The MEMS infrared thin film transducer was installed in a vacuum chamber. The vacuum chamber was connected with a vacuum pump and a refrigerating system. The vacuum working environment (˂5 × 10^−4^ Pa) could greatly reduce the thermal convection between the film surface and air, which could avoid heat loss. The temperature of refrigerating system was not higher than 283 K, which was used to reduce the ambient temperature and suppress the noise. The transducer absorbed the incident visible light, then its temperature increased and the corresponding infrared ray was radiated. An infrared thermal imager collected the infrared ray and presented the infrared image. In the previous work [19], we found that the central wavelength of the radiated infrared ray was correlated to the temperature of the pixels, i.e., the measured central wavelength was about 4.5 µm when the temperature of the pixels reached 473 K, and the whole radiated spectrum covered both middle and long wave infrared region.

## 3. Fabrication and Characterization

### 3.1. Fabrication of the MEMS Infrared Thin Film Transducers

**Figure 6:**
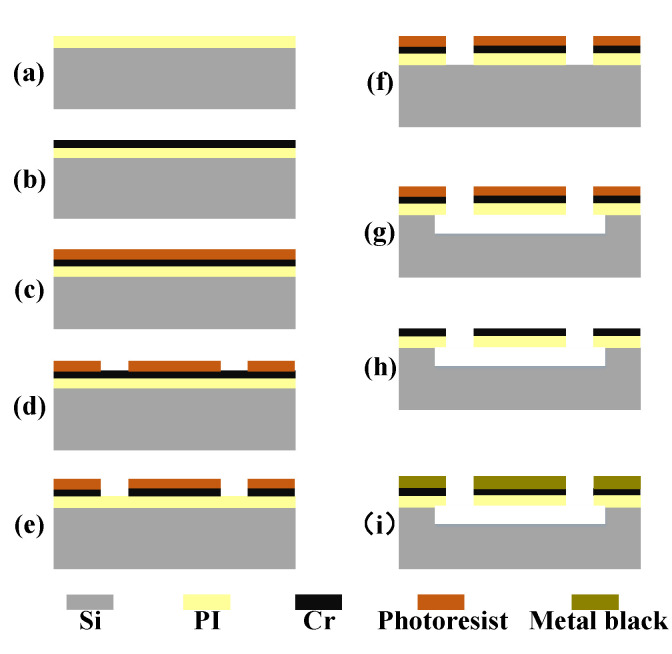
The fabrication process to obtain the proposed robust MEMS infrared thin film transducer with micro cavities: (**a**) PI layer was spin coated; (**b**) Cr film was deposited; (**c**) Photoresist was prepared; (**d**) Photoresist was patterned; (**e**) Cr film was corroded; (**f**) PI layer was etched; (**g**) Silicon cavities were prepared; (**h**) Photoresist was removed; (**i**) Optical absorption layer was evaporated.

Figure 6 illustrates the fabrication procedure of the proposed robust MEMS infrared thin film transducer. First, a 400 nm thick PI layer was spin coated on a 4-inch (100) silicon wafer (Figure 6a). The thickness of the film was controlled by the speed of spin coating. For a 400-nm thick PI layer, the low spinning speed was 800 rpm for 60 s and the high spinning speed was 5000 rpm for 180 s. Then the substrate was placed in an oven and heated at 110 ℃ for 1 h, 150 ℃ for 1 h, and 300 ℃ for 3 h for imidization of PI layer. Next, a chromium (Cr) thin film with 20 nm thickness was deposited on the PI layer as the metal-adhering layer by magnetron sputtering (Figure 6b). The photoresist (S1813 positive photoresist) was prepared on the Cr layer (Figure 6c) by spin coating and UV-lithography was used to pattern photoresist (Figure 6d). To transfer the pattern from photoresist to Cr layer, wet etching was adopted to corrode the Cr layer (Figure 6e). The whole wafer was put into the corrosive solution (99.0% ammonium ceric nitrate: 36% glacial acetic acid:distilled water = 25 g: 20 mL:100 mL) until the pattern on the Cr layer was completely obtained. Then the pattern was transferred down to PI layer by the reactive ion-etching (RIE) process (Figure 6f). The reactant gases were $O_{2}$ and CHF_3_ (15 sccm:30 sccm, 200 W). After that the isotropic etching of silicon was carried out by inductively coupled plasma (ICP) process (Figure 6g) and the reactant gas was SF_6_ (60 sccm, 300 W).

Finally, the photoresist was removed by soaking the wafer in acetone solution (Figure 6h) and the aluminum (Al) black as the optical absorption layer was evaporated and adhered to the Cr layer (Figure 6i). The Al wire (99.999%, 0.5 mm diameter, 6 cm length) was wound on the tungsten wire for thermal evaporation. Thermal evaporation was carried out in helium atmosphere (99.999%) with the pressure of 980 Pa, and the evaporation rate of aluminum was controlled by the current on the tungsten wire. The dimension of the Al black cluster increases with the increase of evaporation rate. The preparation of aluminum black absorption layer can be divided into two steps: a small evaporation rate (33 nm/s) for 300 s, and a large evaporation rate (250 nm/s) for 60 s.

The fabrication procedure of the traditional soft transducer was almost the same as that of the above robust one, other than oxidized silicon wafer was used to replace silicon wafer and there was no need to isotropically etch silicon. In addition, after removing the photoresist, we used hydrofluoric acid (HF) buffer solution to corrode the silicon dioxide sacrificial layer to release the soft composite film and a large PI or silicon ring was used to support the film.

In the fabrication procedure of the robust transducer without silicon micro cavities, isotropic etching of silicon was omitted and the rest parts were the same as in Figure 6.

**Figure 7:**
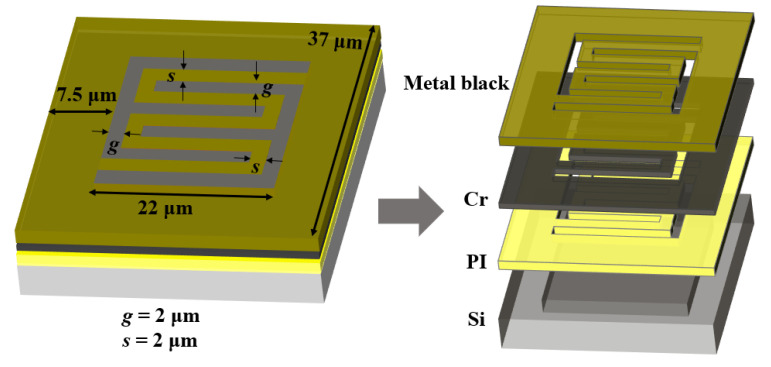
Schematic of a single pixel in the proposed robust MEMS infrared thin film transducer.

Figure 7 presents the detailed configuration of a single pixel in the proposed robust MEMS infrared thin film transducer. To ensure a large temperature difference, a double-S pattern was applied to obtain lower heat transfer efficiency within a limited area. The width of the frame around the double-S pattern also played an important role in the temperature and time response characteristics. We selected the same frame width of 7.5 µm as the reported traditional soft transducer [38] for comparison. The single pixel was a square with a side length of 37 μm, and the inner pixel pattern was a square with a side length of 22 μm. The width of the double-S stripe and gap were both 2 μm. The length of the stripe was 102 μm. When the ICP process was used for isotropic etching, the etchant gas would drill through the 2-μm gap to laterally etch the silicon beneath PI layer. The etching process did not stop until the hollow micro cavity structure was formed.

### 3.2. Characterization of the MEMS Infrared Thin Film Transducer

**Figure 8:**
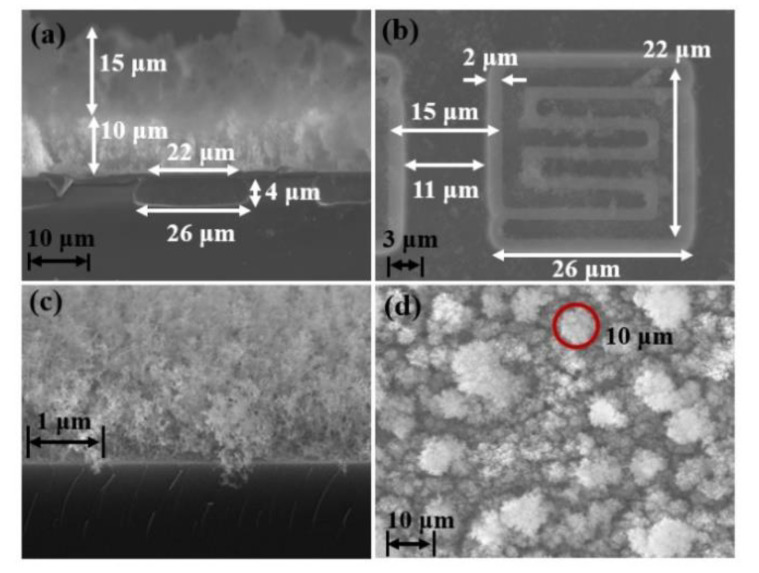
SEM images of the fabricated robust MEMS infrared thin film transducer: (**a**) cross-sectional image of the transducer; (**b**) top view of the pixel; (**c**) bottom optical absorption layer; (**d**) top optical absorption layer.

**Figure 9:**
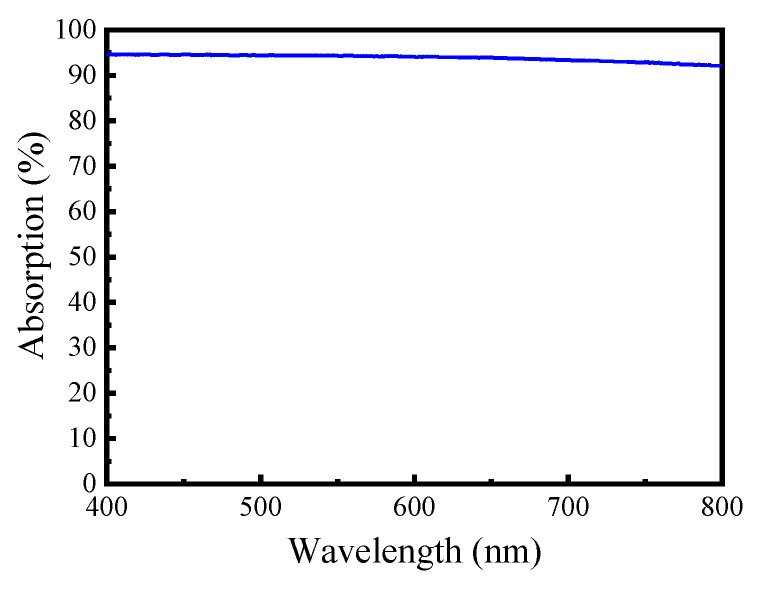
The measured absorption of the optical absorption layer.

The thicknesses of the PI layer and Cr layer were measured by a step profiler (DektakXT, Bruker, Middlesex County, MA, USA). But for the Al black layer, its structure was too loose to stand the probe of step profiler. Its thickness was observed by field emission scanning electron microscope (FE-SEM, Zeiss SUPRA 55 SAPPHIRE, Oberkochen, Germany). The SEM images of the fabricated robust MEMS infrared thin film transducer are shown in Figure 8. The cross-sectional image (Figure 8a) shows that the longitudinal etching depth of the micro cavity is 4 μm and the transversal etching width is 2 μm. The aspect ratio of longitudinal etching to transversal etching is 2:1. From the top view of the pixel (Figure 8b), the width of the silicon supporting wall between adjacent micro cavities is 11 μm. The optical absorption layer is composed of two layers of aluminum black. The bottom layer is around 10 μm thick and it has quite small particles with a dimension of nanometer level (Figure 8c). The top layer is about 15 μm thick and it has much larger particles with a dimension of micrometer level (Figure 8d). Both the two layers exhibit prominent porous cotton-like structure. When the visible light is incident into this porous cotton-like structure, the light inside forms multiple reflections and performs a light-trapping effect. Thus, this porous structure ensures Al black layer has a high absorption efficiency in visible light range. When the diameters of the Al black cluster increase, the effect of light-trapping is enhanced. However, the clusters with large diameters have poor adhesion. To improve the adhesion between large clusters and metal-adhering layer, a bottom layer of Al black clusters with small diameters is applied. The top layer benefits much rougher surface and significantly augment the inside reflection of the visible light. In the infrared transducer, the absorption of visible light is mainly conducted by the optical absorption layer. The absorption efficiency of this optical absorption layer was measured and the result is presented in Figure 9. Because of the cotton-like structure, the incident light is trapped into the clusters and the average absorption is around 95% in the wavelength range of 400 nm to 800 nm.

**Figure 10:**
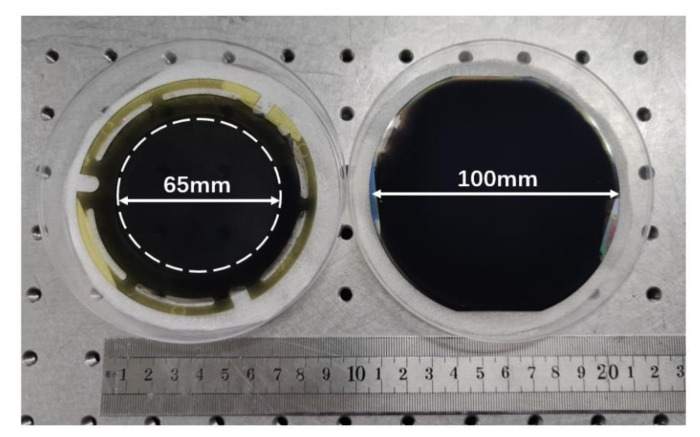
Photo of the traditional soft MEMS infrared thin film transducer (**left**) and the proposed robust MEMS infrared thin film transducer (**right**).

Figure 10 presents the photo of the fabricated traditional soft MEMS infrared thin film transducer (left) and the proposed robust MEMS infrared thin film transducer (right). The traditional soft one has 1313 × 1313 pixels, the same as the reported one [38]. It needs to spare the outer edge to ensure successful attachment to the PI ring of 65 mm diameter. The film becomes more fragile when its diameter becomes larger.

**Figure 11:**
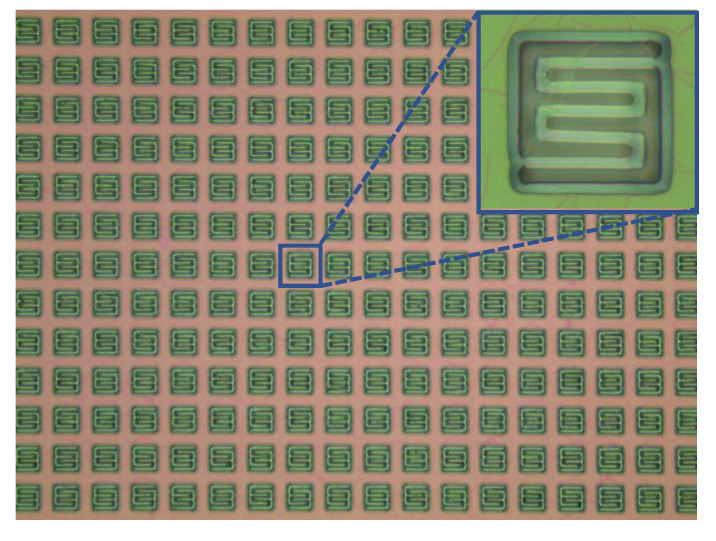
Micrograph of the proposed robust MEMS infrared thin film transducer before the metal black layer was prepared.

Fortunately, the robust transducer does not have the above restriction. The available area can occupy almost the entire surface of the 4-inch silicon wafer. The single pixel is 37 μm × 37 μm. The pixel array scale of the transducer is larger than 2000 × 2000. Figure 11 is its micrograph of the pixel array before the metal black layer was prepared.

**Figure 12:**
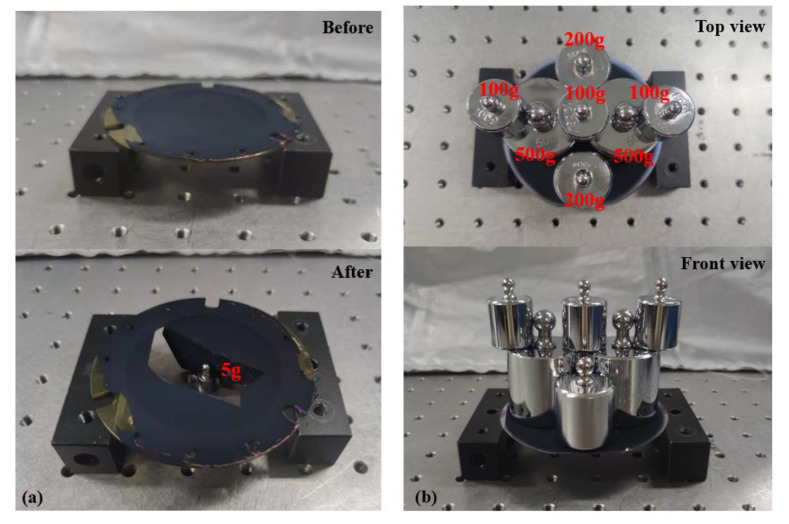
Mechanical stability test of (**a**) the traditional soft MEMS infrared thin film transducer and (**b**) the proposed robust MEMS infrared thin film transducer.

To test the mechanical stability of the traditional soft MEMS infrared thin film transducer and the proposed robust MEMS infrared thin film transducer, the weights were applied and placed on the surface of the two transducers as shown in Figure 12.

In Figure 12a, the weight that the traditional soft MEMS infrared thin film transducer could bear was less than 5 g, and the corresponding surface pressure was 815 Pa. This manifested that the traditional soft MEMS infrared thin film transducer was very fragile and easy to rupture during the installation and use.

For the proposed robust MEMS infrared thin film transducer shown in Figure 12b, it could bear more than 1700 g weight and the maximum pressure exceeded 4275 Pa. The weight that the robust one could bear was more than 340 times of the traditional soft one and the pressure that the robust one could bear was more than 5.24 times of the traditional soft one. Thus, the proposed robust MEMS infrared thin film transducer has excellent mechanical stability compared to the traditional soft transducer, which means it is more robust during the installation and use.

## 4. Simulation and Experiments

### 4.1. Simulation

#### 4.1.1. Simulation Model

A finite element model (FEM) was built to calculate the infrared radiation property. Table 2 shows the parameters of materials in the model. The previous simulation results indicated that the thickness of silicon wafer had no remarkable effect on the radiation characteristics [42]. To reduce the calculating overhead, the thickness of silicon wafer here was selected as 6 µm, and its density, heat capacity and thermal conductivity were 2329 kg/m^3^, 700 J/(kg∙K), and 130 W/(m·K), respectively. These parameters were all set according to the parameters of bulk silicon.

For the PI layer, its thickness, density, heat capacity, and thermal conductivity were set to 0.4 µm, 1350 kg/m^3^, 650 J/(kg∙K), and 0.2 W/(m·K), respectively, which were obtained from the measured results and reference [44]. The Cr adhesion layer was only 20 nm thick, the bottom Al black layer was 10 µm thick, and the top Al black layer was 15 µm thick. This combination of quite thin and quite thick layers led to tremendous mesh and resulted in significant calculation memory and time consumption. To solve this problem, this model applied an equivalent metal layer to substitute the above three layers. The thickness, density, and heat capacity were 10 µm, 350 kg/m^3^, and 127 J/(kg∙K), respectively. Its thermal conductivity *k* was a linear function of the temperature. The equivalent parameters were deduced based on the method in Ref. [38].

#### 4.1.2. Simulation Results

**Figure 13:**
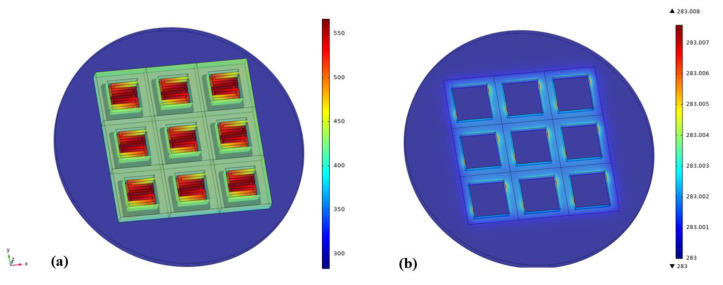
FEM simulation transient results of the proposed robust MEMS infrared thin film transducer at the end of the rising edge of a 50 Hz pulse. (**a**) The surface temperature of the pixels; (**b**) the surface temperature of the silicon layer.

Figure 13 presents the FEM transient simulation results of the proposed robust MEMS infrared thin film transducer at the end of the rising edge of a 50 Hz pulse with 50% duty cycle. It can be seen from Figure 13a, at the end of the rising edge of the 50 Hz pulse, the temperature of the silicon substrate was less than 300 K, and the frame of the pixel that directly contacted the silicon substrate was no more than 450 K. The temperature of the double-S stripe that did not contact the silicon substrate was higher. The joint of the double-S stripe and frame was about 450 K but the temperature in the center of the stripe was above 550 K. Although the input source was a uniform pulse all over the surface of the device, not only on the double-S strip but also on the frame, the temperature distributed non-uniformly because of the structure of the pixels. When the input laser power density increased, the temperature at the center of the double-S strip would also increase. In Figure 13b, the temperature of silicon micro cavities was around 283 K. Although the temperature difference was quite small (<0.01 K), the temperatures at different positions of the microcavities were different. The maximum temperature of silicon micro cavities was at the contact area with the frame of the pixels. This result indicated that the heat dissipation rate of silicon substrate was relatively fast compared to that of the pixel array. It was because that the thermal conductivity of silicon (130 W/(m∙K) [40]) was 650 times of that of PI (0.2 W/(m∙K) [38]) and about 5200 times of metal layer (˂0.025 W/(m∙K) [38]) as shown in Table 2. The micro cavities contacted with the frame played an important role in heat conduction.

**Figure 14:**
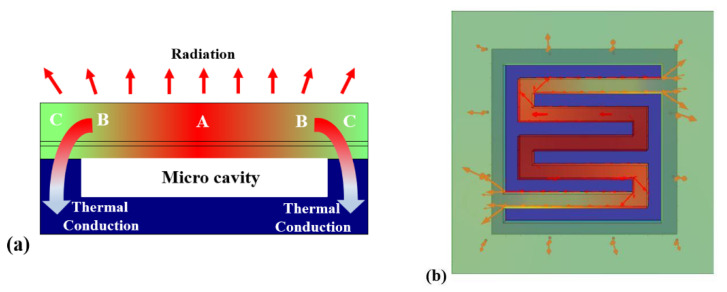
Heat flux distribution of the proposed robust MEMS infrared thin film transducer. (**a**) Schematic of heat flux distribution in thickness direction; (**b**) FEM simulation result of heat flux distribution in plane direction.

Figure 14 shows the heat flux distribution of the proposed robust MEMS infrared thin film transducer. Figure 14a is the schematic of the heat flux distribution in thickness direction. As aforementioned in Equation (1), heat flux was transferred mainly by heat conduction and radiation in vacuum environment. In three-dimensional conduction, most heat flux was transferred through the joint area of the frame and silicon. Figure 13b also proves this phenomenon as the maximum temperature of silicon micro cavity was located in the connection area. Therefore, in the area of single pixel, the highest temperature was located in Region A, and the temperature of Region B was higher than that of Region C, as shown in Figure 14a.

Figure 14b presents the heat flux in the central double-S stripe spread out through the connecting legs on both sides of the frame. As the frame was connected with the silicon layer whose thermal conductivity was quite high, the frame area dissipated heat fastest and its temperature was lowest compared with the suspended double-S stripe. Conduction was the main way of heat transfer compared to radiation, thus, the temperature at the center of double-S stripe was higher than at the connecting legs and the frame.

**Figure 15:**
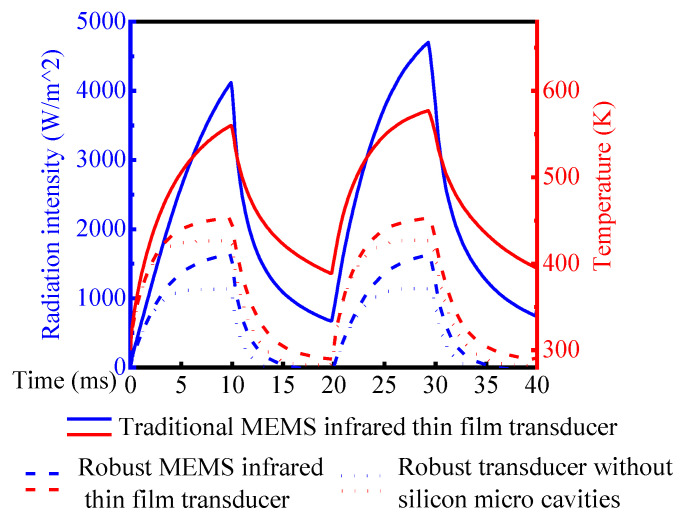
The transient simulation results of the three MEMS infrared thin film transducers.

Figure 15 illustrates the transient simulation results of the three kinds of MEMS infrared thin film transducers. The temperature and radiation intensity data were the surface average values of the pixel array. Thermal decay time was defined as the duration of the radiation intensity decreasing from the maximum to zero. Thermal decay time was inversely proportional to frame rate. Hence, the frame rate got higher when the thermal decay time got shorter. The thermal decay time of the proposed robust MEMS infrared thin film transducer and robust transducer without silicon micro cavities was 6.0 ms and 2.5 ms, and their radiation intensities were 1628 W/m^2^ and 1140 W/m^2^. The thermal decay time of the two transducers was smaller than half cycle of 50 Hz (10 ms), therefore there was little heat accumulation in a cycle and the radiation intensity deceased to zero before the next new cycle. However, the thermal decay time of traditional soft MEMS infrared thin film transducer was obviously more than 10 ms and the radiation intensity decreased to 830 W/m^2^ when the next new cycle just began. Compared to the other two transducers, this traditional soft transducer did not have a large conductivity material (Si) as the substrate, so its heat dissipation was slow and that led to heat accumulation. The heat accumulation of traditional soft transducer resulted in the next cycle’s maximum radiation intensity (4700 W/m^2^) higher than that of the previous cycle (4100 W/m^2^). The temperature curve had the similar characteristic with the radiation curve.

The comparison and evaluation of the three transducers are shown in Table 3.

It can be seen from Table 3 that the merit and demerit of the traditional soft MEMS infrared thin film transducer and the robust MEMS infrared thin film transducer without micro cavities were both prominent. Among the three transducers, the radiation and temperature characteristics of the traditional soft one were good but the frame rate was poor. The robust transducer without micro cavities had the opposite properties.

The proposed robust MEMS infrared thin film transducer had the advantages from both sides and kept a more desired balance. The thermal decay time of the proposed robust infrared thin film transducer was reduced by at least 50% than that of the traditional soft one, and its radiation intensity was increased by 42.8% than that of the robust transducer without micro cavities.

### 4.2. Experiments

#### 4.2.1. Experimental Setup

**Figure 16:**
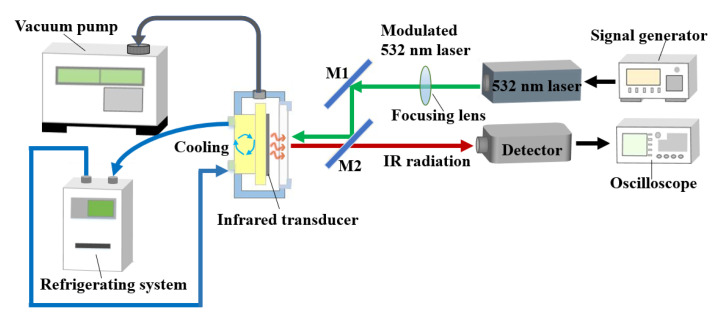
Schematic of experimental setup for measuring time property of the fabricated MEMS infrared thin film transducer.

The infrared radiation property is an important index to characterize the infrared transducers. Generally, the radiation property includes spatial property and time property. The radiation spatial property consists of spatial resolution, contrast, etc. The radiation time property consists of time response of radiation intensity and temperature. We tested the radiation spatial property using the experiment setup in Figure 5, and obtained the radiation time property using the setup in Figure 16.

In the experiment of radiation spatial property, the input source was provided by a projector (BenQ, MX535, 50~60 Hz), and the infrared images were obtained by thermal imager (InfraTec, VarioCAM HD head 680, 7.5~14 μm).

In the experiment of time property, the fabricated MEMS infrared thin film transducer was installed in a vacuum chamber. Differently from that in Figure 5, the light source was a 532 nm laser signal rather than a projector. The laser power density was 5.4 W/cm^2^. A 50 Hz square wave signal with 50% duty cycle was given to the laser through a signal generator. The modulated laser was focused by a lens and reflected by an optical reflector M1 and an optical mirror M2 and then incident to a BaF_2_ window of the vacuum chamber. The transducer absorbed the incident light, then its temperature increased and the corresponding infrared ray was radiated. The radiated infrared ray transmitted through the BaF_2_ window and the optical mirror M2 which reflected the visible light (400~800 nm) and transmitted infrared light (3~12 μm). An infrared point source detector (PVI-4TE-5, VIGO System S.A., working band covers 2~13 μm) collected the infrared ray, and an oscilloscope was utilized to record the output signals.

#### 4.2.2. Measurement Results

**Figure 17:**
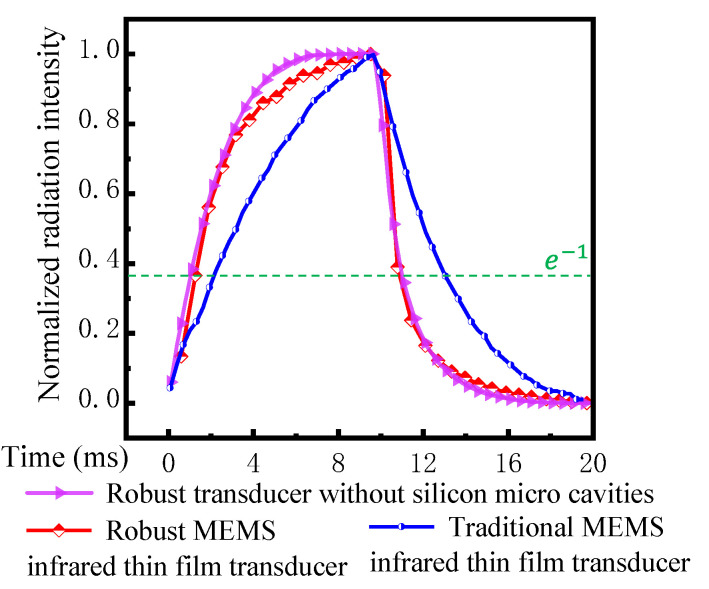
Measured results of normalized radiation intensity for the three MEMS infrared thin film transducers.

Figure 17 presents the measured results of normalized radiation intensity for the three transducers. The time constant was defined as the duration of the radiation intensity decreasing from the maximum to e^−1^ of the maximum. The time constants of traditional soft MEMS infrared thin film transducer, the proposed robust MEMS infrared thin film transducer, and the robust transducer without silicon micro cavities were 3.55 ms, 1.75 ms, and 1.01 ms, respectively. It indicated that the introduction of silicon substrate greatly improved the time response. The time constant of the proposed robust MEMS infrared thin film transducer was 50.7% smaller than the traditional soft transducer. The robust transducers had comparable time constants despite of the difference in micro cavities.

**Figure 18:**
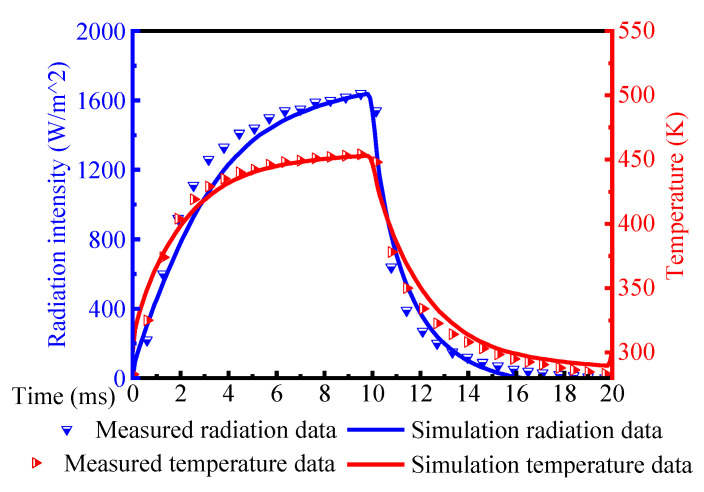
Transient simulated and measured results of the proposed robust transducer with micro cavities.

To obtain both the time response and radiation characteristics, we used a thermal imager to acquire the transient temperature and radiation intensity of the proposed robust transducer. Figure 18 illustrates the simulated and measured results of the proposed robust transducer with micro cavities. In the measured condition, the maximum radiation intensity was 1600 W/m^2^, and the temperature difference reached 171 K (from 283 K to 454 K). The highest temperature of 454 K covered the radiated spectrum of both middle (3–5 μm) and long wave (8–12 μm) infrared [19].

The measured curves were well consistent with the simulation results. The slight difference between them may be due to the number of pixels. In the simulation model, 3 × 3 pixels were selected in order to save calculation time while the measured transducer had more than 2000 × 2000 pixels.

**Figure 19:**
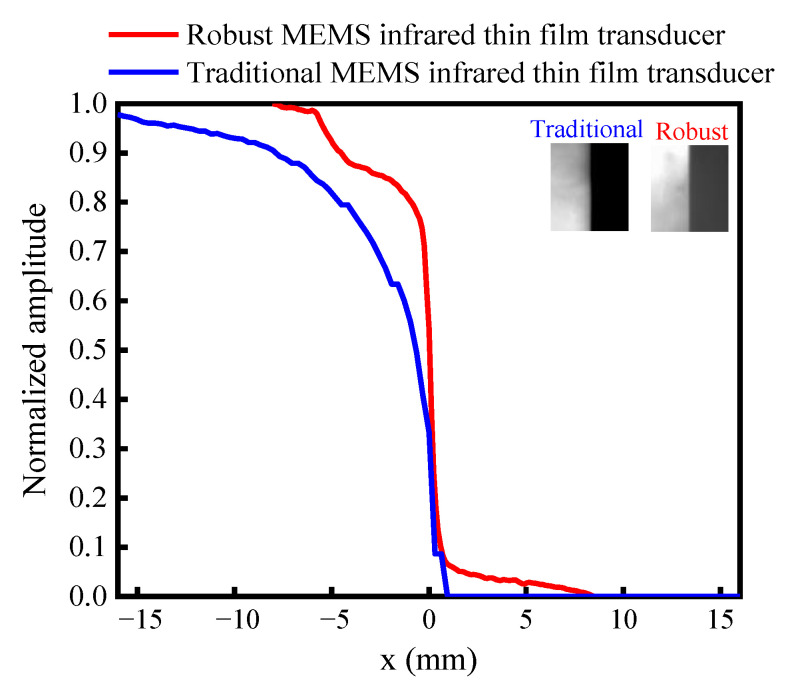
The edge spread function of the traditional soft MEMS infrared thin film transducer and the proposed robust MEMS infrared thin film transducer.

**Figure 20:**
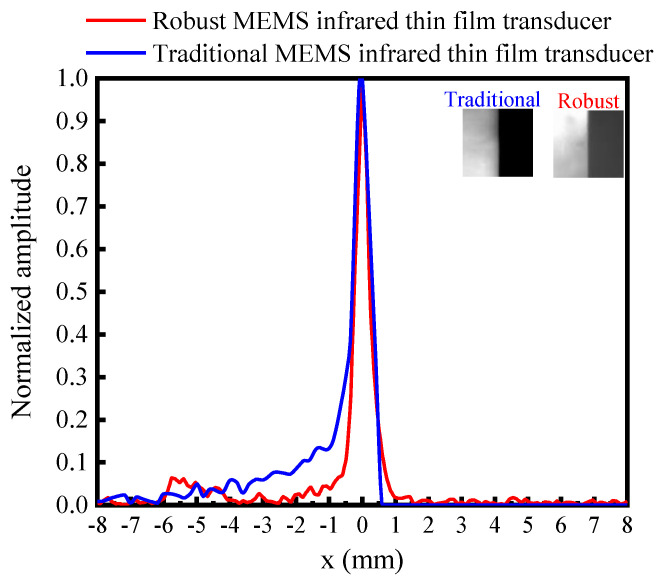
The line spread function of the traditional soft MEMS infrared thin film transducer and the proposed robust MEMS infrared thin film transducer.

We used the experiment setup in Figure 5 to compare the imaging quality. A knife edge image was projected to the traditional soft MEMS infrared thin film transducer and the proposed robust transducer by the visible projector. Figure 19 is the edge spread function obtained by normalizing the gray value of the output image. It indicated the proposed robust transducer had a sharper slope than the traditional one. For the traditional soft transducer, the intensity in the bright region varied more dramatically than that in the dark region. While the proposed robust transducer possessed a symmetrical distribution on both sides. Figure 20 is the line spread function from the derivation of the edge spread function. The proposed robust one had a FWHM (full width at half maximum) of around 470 μm, while the traditional soft one had a FWHM of about 620 μm. Therefore, the proposed robust one had a spatial resolution 24% higher than the soft one. The previously reported result presented that the measured spatial resolution of DMD infrared transducer was 217 μm [21], 53.8% better than our proposed robust one. Nevertheless, the proposed robust one would be improved greatly in spatial resolution if the thermal crosstalk and the pattern of the pixel be optimized. Future work will include the thermal isolation and structural optimization of the proposed robust transducer to reach superior performances, such as spatial resolution, radiation intensity, time response, etc.

**Figure 21:**
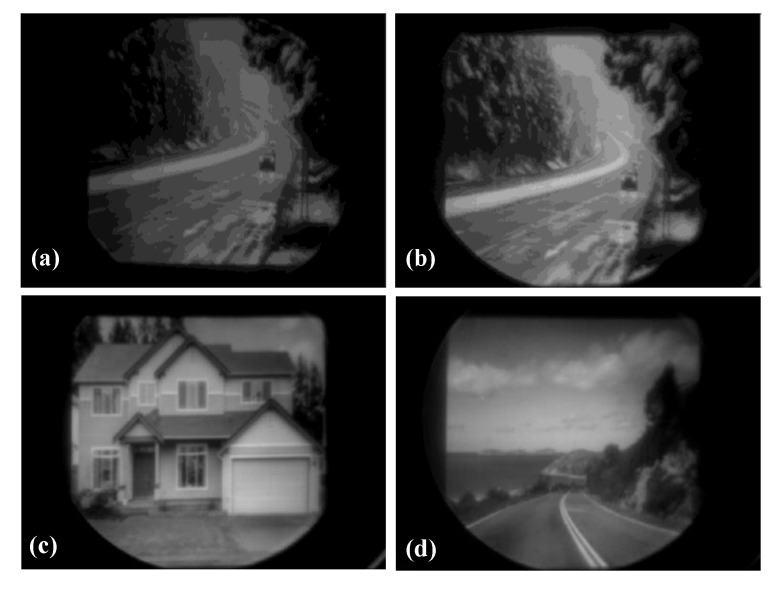
The infrared images generated by (**a**) the traditional soft MEMS infrared thin film transducer and (**b**–**d**) the proposed robust MEMS infrared thin film transducer.

Figure 21 displays the infrared images generated by the traditional soft MEMS infrared thin film transducer (Figure 21a) and the proposed robust transducer (Figure 21b–d). The image generated by the proposed robust transducer had a higher resolution than the soft one, which was consistent with the measured result as illustrated in Figure 20.

#### 4.2.3. Comparison and Discussion

Table 4 presents the comparison of the traditional soft MEMS infrared thin film transducer and the proposed robust infrared thin film transducer.

Although the radiation intensity and temperature difference of the traditional soft MEMS infrared thin film transducer were higher than the proposed robust transducer, its time response and spatial resolution could not surpass that of the proposed robust one. Moreover, the traditional soft MEMS infrared thin film transducer had heat accumulation in high frame rate, which was undesired for the infrared dynamic images or videos.

The full width at half maximum (FWHM) of the proposed robust MEMS infrared thin film transducer was 24% smaller than the traditional soft transducer. The time constant of the robust transducer decreased by 50.7% compared to the traditional soft one. More importantly, compared to the traditional soft transducer, the mechanical stability of the robust transducer had a great improvement and the load pressure was improved by more than 5.24 times. The pixel number of the propose robust infrared thin film transducer was more than 2000 × 2000, thus the array scale was enlarged by more than 52.3% compared to the traditional soft infrared thin film transducer.

## 5. Conclusions

A robust MEMS infrared thin film transducer beyond 2000 × 2000 pixels was proposed and fabricated on a 4-inch silicon wafer. Compared with the traditional soft infrared thin film transducer, the load pressure of the proposed robust transducer was improved by more than 5.24 times and time constant decreased by 50.7%. The proposed transducer could bear a load of 1700 g, while the soft one ruptured under a load of 5 g. Radiation time property experiment demonstrated that the introduction of silicon substrate greatly improved the time response and the silicon cavities could regulate the rate of heat conduction. Thermal decay time of the robust transducer was 6.0 ms when the input 532 nm laser had a frequency of 50 Hz with 50% duty cycle. The simulation and measured results were well consistent. These results indicated that this novel transducer had excellent mechanical stability, time response, and state-of-the-art pixel scale. Full width at half maximum (FWHM) of the robust MEMS infrared thin film transducer was 24% smaller than the traditional soft transducer in radiation spatial property test. High resolution infrared images were generated using the robust transducer. It proved that the robust transducer was promising in infrared image generation. This will greatly facilitate the development of infrared target recognition in unmanned driving technology.

## Figures and Tables

**Table 1:** Comparison of widely used infrared image generation technologies.

Parameter	DMD	Resistor Array	MEMS Infrared Thin Film Transducer
Radiation wavelength range (µm)	mid and longwave infrared bands (poor in longwave infrared band)	mid and longwave infrared bands	**mid and longwave infrared bands**
Array size	1280 × 1024	1024 × 1024	**1313 × 1313**
Pixel size (µm)	13	48	**35**
Frame rate	690 Hz	200 Hz	50 Hz
Read in circuit	complicated	complicated	**no need**
Reference	[20,21,31,32]	[14,15,16]	[17,18,19]

**Table 2:** Parameters of materials used in FEM simulation [38,43,44].

Parameter	Si	PI	Metal
Thickness (µm)	6	0.4	10
Density (${\mathrm{kg}/m}^{3}$)	2329	1350	350
Constant pressure heat capacity (J/(kg⋅K))	700	650	127
Thermal conductivity (W/(m⋅K))	130	0.2	*K* *

* The measured parameter is presented in Appendix A.

**Table 3:** Comparison and evaluation of the three transducers on transient simulation results.

Parameter	Traditional Soft MEMS Infrared Thin Film Transducer	Proposed Robust MEMS Infrared Thin Film Transducer	Robust MEMS Infrared Thin Film Transducer without Micro Cavities
Radiation intensity (W/m^2^)	4100 (first cycle)	1628	1140
Maximum temperature (K)	560 (first cycle)	450	426
Thermal decay time (ms)	>10	6	2.5
Heat accumulation	poor	good	good
Comprehensive assessment	The radiation and temperature characteristics were good but the frame rate was poor	The radiation and temperature characteristics were medium and the frame rate was medium	The radiation and temperature characteristics were poor but the frame rate was good

**Table 4:** Comparison of the proposed robust infrared thin film transducer and traditional soft MEMS infrared thin film transducer.

Parameter	Traditional Soft MEMS Infrared Thin Film Transducer	Robust Infrared Thin Film Transducer
Array size	1313 × 1313	more than 2000 × 2000
Time constant (ms)	3.55	1.75
50 Hz frame rate	medium	good
FWHM (μm)	620	470
Radiation intensity and temperature difference	high	medium
Mechanical stability	poor	good

**Figure A1:**
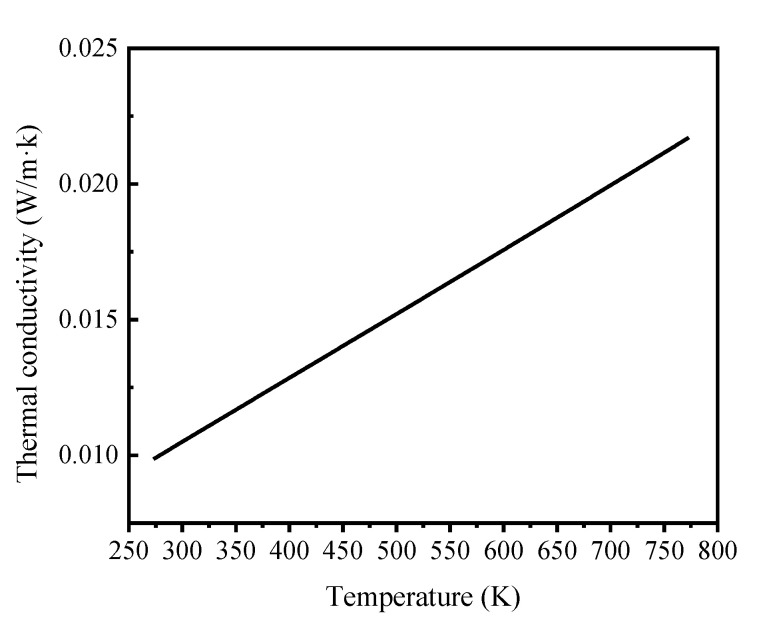
Thermal conductivity of the equivalent metal layer.

## Appendix A

*K* * is the thermal conductivity of the equivalent metal layer. Figure A1 presents the curve of thermal conductivity changing with temperature measured by the method in Ref. [22].
